# ‘Efficacy and safety outcomes after slings or artificial urinary sphincter implantation for non-neurogenic male stress urinary incontinence: a systematic review’ for more clarity

**DOI:** 10.3389/fruro.2026.1852334

**Published:** 2026-10-02

**Authors:** Cyrille Guillot-Tantay, Sabrina de Cillis, Paolo Geretto, Luis Vale, Manuela Tutolo, Fabiana Cancrini, Véronique Phé, Nadir I. Osman, Mikolaj Przydacz

**Affiliations:** 1 Hôpital Foch, Service d’urologie et de transplantation rénale, Suresnes, France; 2 Department of Oncology, Division of Urology, University of Turin, San Luigi Gonzaga Hospital, Orbassano, Italy; 3 Division of Neuro-Urology, Department of Surgical Sciences, CTO Hospital, Citta della Salute e della Scienza, Turin, Italy; 4 Department of Urology, Centro Hospitalar Universitario Sao Joao, Porto, Portugal; 5 Unit of Urology, Division of Oncology, Urological Research Institute, IRCCS San Raffaele Hospital, Milan, Italy; 6 Department of Medical and Surgical Sciences and Translational Medicine, Sapienza University, Rome, Italy; 7 Department of Urology, Sorbonne University, Assistance Publique-Hôpitaux de Paris, Tenon Academic Hospital, Paris, France; 8 Department of Urology, Royal Hallamshire Hospital, Sheffield, United Kingdom; 9 Department of Urology, Jagiellonian University Medical College, Krakow, Poland

**Keywords:** artificial urinary sphincter, medical devices, mid-urethral sling, systematic review, urinary incontinence

## Abstract

**Introduction:**

Our objective was to conduct a systematic review of studies comparing male slings (MS) and Artificial Urinary Sphincter (AUS) in terms of efficacy and safety in men with Stress Urinary Incontinence (SUI).

**Methods:**

We conducted a systematic review including all studies comparing MS and AUS in adult men with SUI. All synthetic MS were included. The primary outcome was the postoperative continence rate defined as either no pad use or one safety pad.

**Results:**

Of 133 references screened, after exclusion of duplicates, 18 papers were eligible for a full-text analysis and 9 studies (5921 patients) were included in the qualitative synthesis. Among these, one was a randomized controlled trial and eight were cohort studies. The cure rate varied from 14% to 94% for the AUS and 11% to 69% for the MS. Of the five studies reported both devices, two (40%) reported a continence rate above 70% for AUS. Eight of the nine studies (88.9%) reported safety outcomes. Urethral erosion was less frequent after male slings than after artificial urinary sphincter implantation, whereas urinary retention was more frequent after male slings in most studies.

**Conclusion:**

In comparative studies, continence proportions were generally higher after artificial urinary sphincter implantation, while the complication profiles of the two devices differ, with more erosion after the sphincter and more urinary retention after the sling. Confidence in these findings is limited by clinical heterogeneity, confounding by indication and inconsistent outcome definitions. Standardised outcome reporting and randomised data are needed.

**Systematic review registration:**

https://www.crd.york.ac.uk/prospero/, identifier CRD42024539539.

## Introduction

Stress Urinary Incontinence (SUI) is defined by the International Continence Society as “the complaint of involuntary loss of urine on effort or physical exertion including sporting activities, or on sneezing or coughing” (1). In men, it most frequently occurs after radical prostatectomy (RP) due to either direct injury to the external urethral sphincter or the neurovascular bundles, shortening of the membranous urethra or displacement of the sphincter due to loss of posterior support. The reported incidence of SUI following RP has varied widely, from 5% to 48%, probably explained by variations in technique, definitions used and timing of evaluation. Despite pelvic floor muscle training, SUI persists in 5-10% of men 12 months after RP, and surgical treatment is often considered (2). Among the different surgical options, the artificial urinary sphincter (AUS) introduced in the 1970’s by Scott, is highly effective and is widely regarded as the gold standard surgical treatment (2).

The male sling (MS), first introduced by Kaufman in the 1970s initially failed to gain favor due to high rates of urinary retention and urethral erosion (3, 4). Since then, several modifications in materials, fixation methods, and positioning have been introduced. Advantages of the MS over the AUS include the absence of mechanical parts, no need for device manipulation to void, and lower costs. Several types of MS exist for the management of mild to moderate urinary incontinence, including adjustable, transobturator, and retropubic slings (5). It consists of a polypropylene mesh placed retrourethrally under the proximal urethral bulb. Its mechanism of action is thought to involve compensation for post-prostatectomy laxity of the posterior supporting structures by realigning the sphincter complex toward the pre-prostatectomy configuration.

In recent years, MS have gained popularity due to the ease of surgery, favorable functional results, and low complications rates (6). However, with the increase in salvage radiotherapy for advanced prostate cancer, fewer MS are implanted, favoring AUS, since radiated patients are at higher risk of complications with MS.

Several studies comparing AUS and MS reported that AUS implantation significantly outperformed MS for both subjective and objective outcomes. More evidence from systematic reviews and Randomized Clinical Trials (RCT) is required (7). Despite the growing body of AUS data, there remains a gap in systematic reviews comparing the two surgical modalities. Therefore, our objective was to conduct a systematic review of studies comparing MS and AUS in terms of efficacy and safety in men with stress urinary incontinence (SUI), with the aim of providing clinically useful input for practice.

Systematic reviews comparing artificial urinary sphincters and male slings have been published, but most have pooled single-arm proportions from separate case series, have been restricted to post-prostatectomy incontinence or to moderate incontinence, or have reported efficacy more completely than device-related morbidity. We therefore conducted a systematic review restricted to head-to-head comparative studies, in which both devices were evaluated within the same cohort and with the same outcome criteria, in adult men with non-neurogenic stress urinary incontinence of any aetiology, and we report within-study comparative estimates for continence and for major device-related complications.

## Materials and methods

This systematic review was reported in accordance with the PRISMA 2020 statement (completed checklist in **Supplementary Appendix B**) and the protocol was registered in PROSPERO (CRD42024539539) (8). A completed AMSTAR form is provided as an author self-audit in **Supplementary Appendix A** (9).

### Data sources

A literature search in Medline (via PubMed) and the Cochrane Central database, was conducted from January 1998, when the first MS was described to September 2025 and updated in August 2026, without language restrictions. Only comparative studies were included. We developed a search algorithm (reported in **Supplementary Appendix C**) including keywords and free-text words around: *stress urinary incontinence*, *slings, artificial urinary sphincter* AND *male.*

Conference abstracts of scientific societies (European Association of Urology, American Urological Association and International Continence Society) using the aforementioned keywords were screened. We also searched ClinicalTrials.gov for ongoing or completed studies. Finally, we screened reference lists of relevant studies for any additional reference.

### Eligibility criteria

#### Study types and patient characteristics

All comparative retrospective or prospective studies of MS and AUS in adult men with SUI were included. Case reports, pediatric and female populations, neurogenic patients, cohorts of fewer than five men, cadaveric/animal studies were excluded. Abstracts reporting relevant data were considered. When more than one study reported results from the same cohort, the most recent paper was included. We considered short (up to 1 year), mid (1–5 years) and long-term (>5 years) follow-up studies.

#### Intervention types

All MS and AUS procedures were considered.

#### Outcomes

The primary outcome was continence, defined *a priori* as no pad use or a single safety pad per day. When a study reported both a strict (“cure”, “dry”) and a broader (“improved”, “success”) category, only the strict category was used. The terms “cure”, “success” and “improvement” were not treated as interchangeable.

Secondary outcomes included major device-related complications requiring reoperation:

- Device infection requiring explantation
- Urethral erosion requiring explantation
- Urinary retention requiring explantation

### Selection process

Two authors (CGT and MP) independently screened all citations based by title and abstract, and when necessary, by full text. A third researcher (NO) resolved disagreements.

### Data extraction

Two review authors (CGT and MP) independently extracted:

1. Study design (randomized or not, prospective or retrospective cohort, single or multicenter)
2. General characteristics (year, journal, setting, funding)
3. Patient characteristics (age, follow-up, cause of SUI (RP, radiotherapy, Benign Prostate Hypertrophy - BPH)
4. Surgeries details (MS or AUS)

### Risk of bias assessment

Risk of bias (10) was assessed with the Critical Appraisal Skills Programme (CASP) checklist for cohort studies (11) and the Cochrane ROB tool v2 for RCTs (12).

### Analysis

Studies were described by general, patient and intervention characteristics. Missing data were not imputed.

For each study and each outcome, the risk ratio (RR) of AUS versus MS were computed with 95% confidence intervals. Study-level comparative estimates are displayed as forest plots on a logarithmic scale. Analyses were performed with R version 4.4.1 (meta and metafor packages).

Subgroups analyses were planned but meta-analysis was not performed due to heterogeneity.

The synthesis was a structured narrative synthesis in which studies were grouped by outcome and ordered by effect direction and precision. Pooling was pre-specified as inappropriate when studies differed in study design, outcome definition or follow-up duration.

## Results

### Literature search

The PRISMA flowchart is shown in **Figure 1**. Of 133 references screened, after exclusion of duplicates, 18 papers were eligible for a full-text review, and 9 studies (5921 patients) were included for qualitative synthesis. Publication years ranged from 2009 to 2021. No relevant conference abstracts were included.

**Figure 1:**
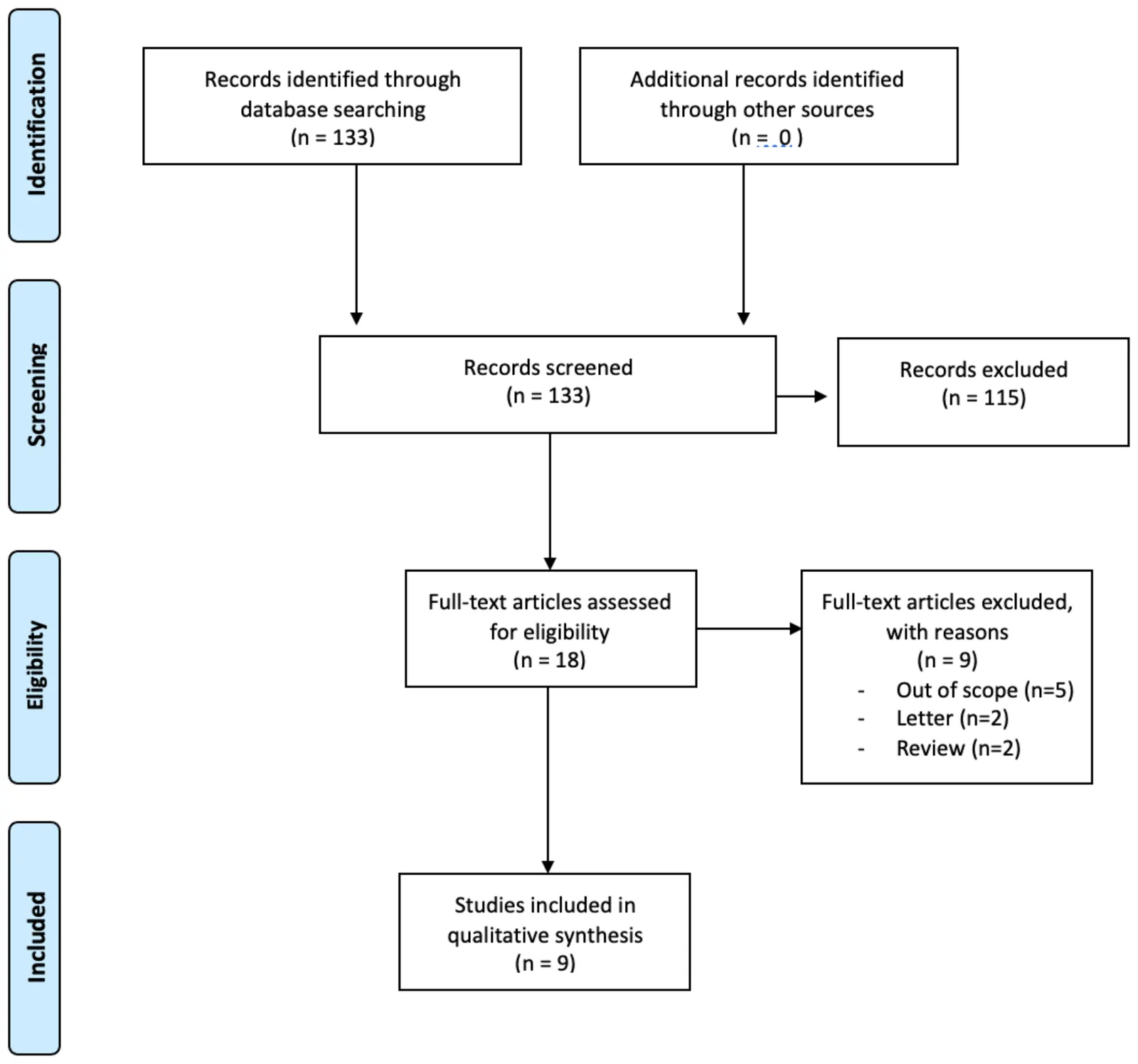
Flow chart of the selection process.

### Study and population characteristics

Of the 9 studies, one was a RCT and eight were retrospective cohorts. Six were multicenter and six were conducted in Europe. Efficacy was the primary outcome in five studies (55.5%).

The median number of patients per study was 176 (35–513) for MS and 190 (35–506) for AUS. Mean follow-up ranged from 1 to 51 months. Mean age ranged from 64–70 years (MS) and 65–70 years (AUS).

Median numbers of patients by etiology were:

- Radical prostatectomy: 157 (73–316) MS vs. 180 (81–390) AUS
- Radiotherapy: 38 (22–48) MS vs. 39 (26–145) AUS
- BPH surgery: 4 (0–12) MS vs. 5 (0–19) AUS

The details of the studies and population are reported in **Table 1**.

**Table 1:** Details of the studies and population.

Study	Year	Design	Centers (N)	Place	Follow-up (m)	Outcomes	N		AGE								INDICATION					
									MS				AUS				MS			AUS		
							MS (N = 2491)	AUS (N = 3430)	M	SD	m	IQR	M	SD	m	IQR	RP (N)	Rx (N)	BPH (N)	RP (N)	Rx (N)	BPH (N)
Abrams (22)	2021	RCT	27	UK	12	Efficacy	190	190			68	64-71			69	63-72	178	38	8	180	39	9
Ajay (23)	2015	C	1	USA	5	Efficacy	29	32	64	6			65	7			29	5	0	32	15	0
Alwaal (24)	2016	C	ND	USA	1	Safety	597	608									ND	ND	ND	ND	ND	ND
Dosanjh (25)	2019	C	48	UK	38	ND	816	1414			68	63-71			68	64-72	816	55	0	1414	146	0
Grabbert (26)	2018	C	13	D	16	Efficacy	176	482	69.9				70				157	40	ND	389	144	ND
Hush (27)	2016	C	ND	D	14	ND	513	506	69.8	6.7			70.4	7.8			474	81	35	403	153	61
Kretshmer (28)	2017	C	13	D		Safety	127	155	70	6.9			69.4	10.9			110	35	14	127	36	24
Sacco (29)	2020	C	1	I	51	Efficacy	35	35			70	65-74			71	67-74	35	8	0	35	9	0
Tuygun (30)	2009	C	1	TK	10	Efficacy	8	8	70				69				ND	ND	ND	ND	ND	ND

MS, Male Slings, AUS, Artificial Urinary Sphincter, RCT, Randomized Controlled Trial, C, Cohort study, UK, United Kingdom, USA, United States of America, D, Germany, I, Italy, TK, Turkey, M, Mean, SD, Standard Deviation, m, median, IQR, Inter Quartile Range, RP, Radical Prostatectomy, Rx, Radiotherapy, BPH, Benign Prostate Hypertrophy, ND, Not Define.

Baseline incontinence severity was reported by arm in a minority of studies and with heterogeneous metrics (daily pad count, 24-hour pad weight test, or descriptive categories). Prior radiotherapy was reported in seven studies and previous continence surgery in five. In every study reporting these variables, men treated with AUS had more severe baseline incontinence and/or more frequent prior radiotherapy than men treated with MS. Only one comparison was randomized and one was propensity-score matched; all other comparisons are unadjusted.

### Risk of bias assessment

Risk of bias assessments are reported in **Supplementary Appendix D** (CASP) and **Supplementary Appendix E** (ROB v2). For cohort studies, risk was generally low, with concerns mainly about follow-up duration. For the RCT, outcome measurement was not blinded.

### Efficacy

Global cure rates (0–1 safety pad/day) are shown in **Figure 2**. Five studies reported cure rates for both AUS and MS. Overall, rates were higher with AUS (14–94%) compared with MS (11–69%). Two studies (40%) reported cure rates above 70% with AUS, compared with none for MS.

**Figure 2:**
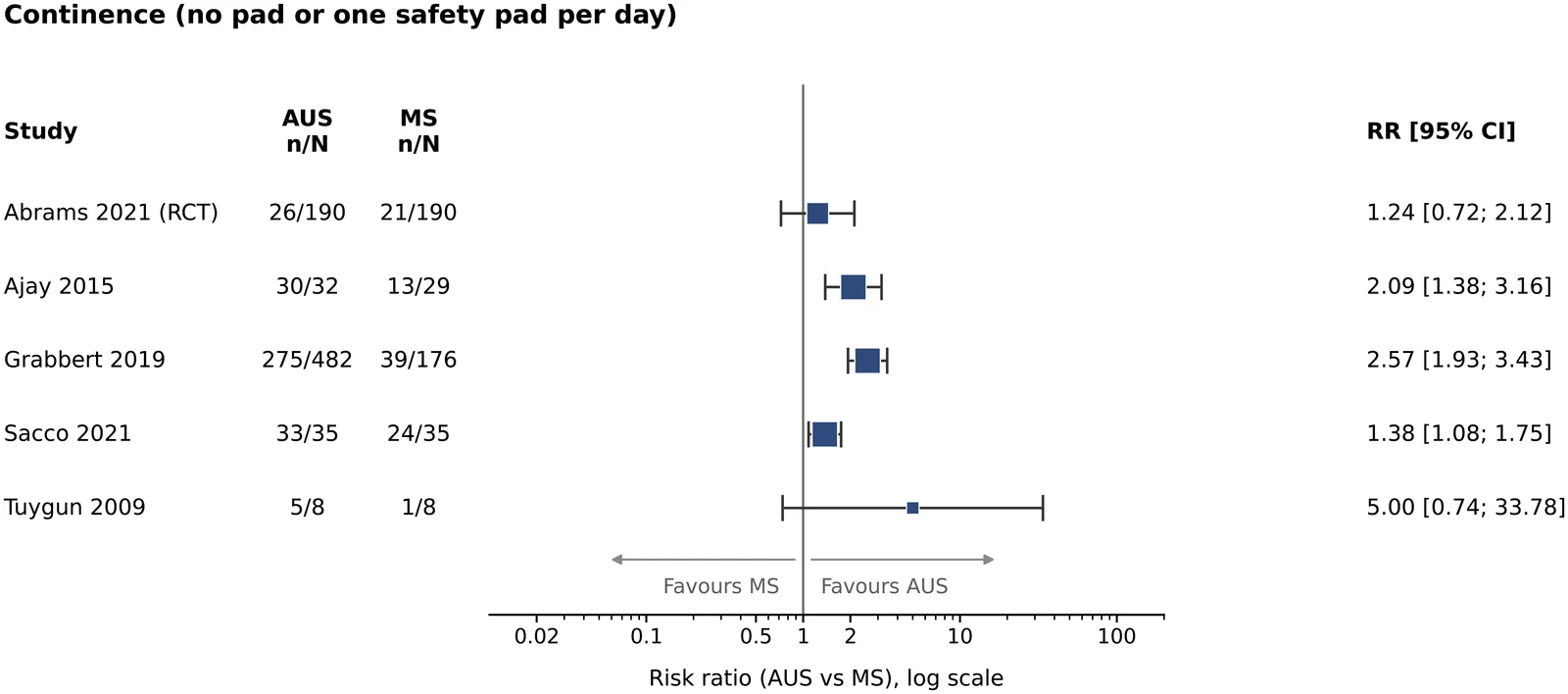
Continence (no pad or one safety pad per day).

The risk ratio of continence for AUS versus MS was above 1 in all five studies: 1.24 (95% CI 0.72–2.12) in the only randomized trial, and 1.38 (1.08–1.75), 2.09 (1.38–3.16), 2.57 (1.93–3.43) and 5.00 (0.74–33.78) in the retrospective cohorts. The difference was statistically significant in three of the five studies, all of them retrospective cohorts.

### Safety

Safety data are shown in **Figure 3**. Eight studies (88.9%) reported safety outcomes:

**Figure 3:**
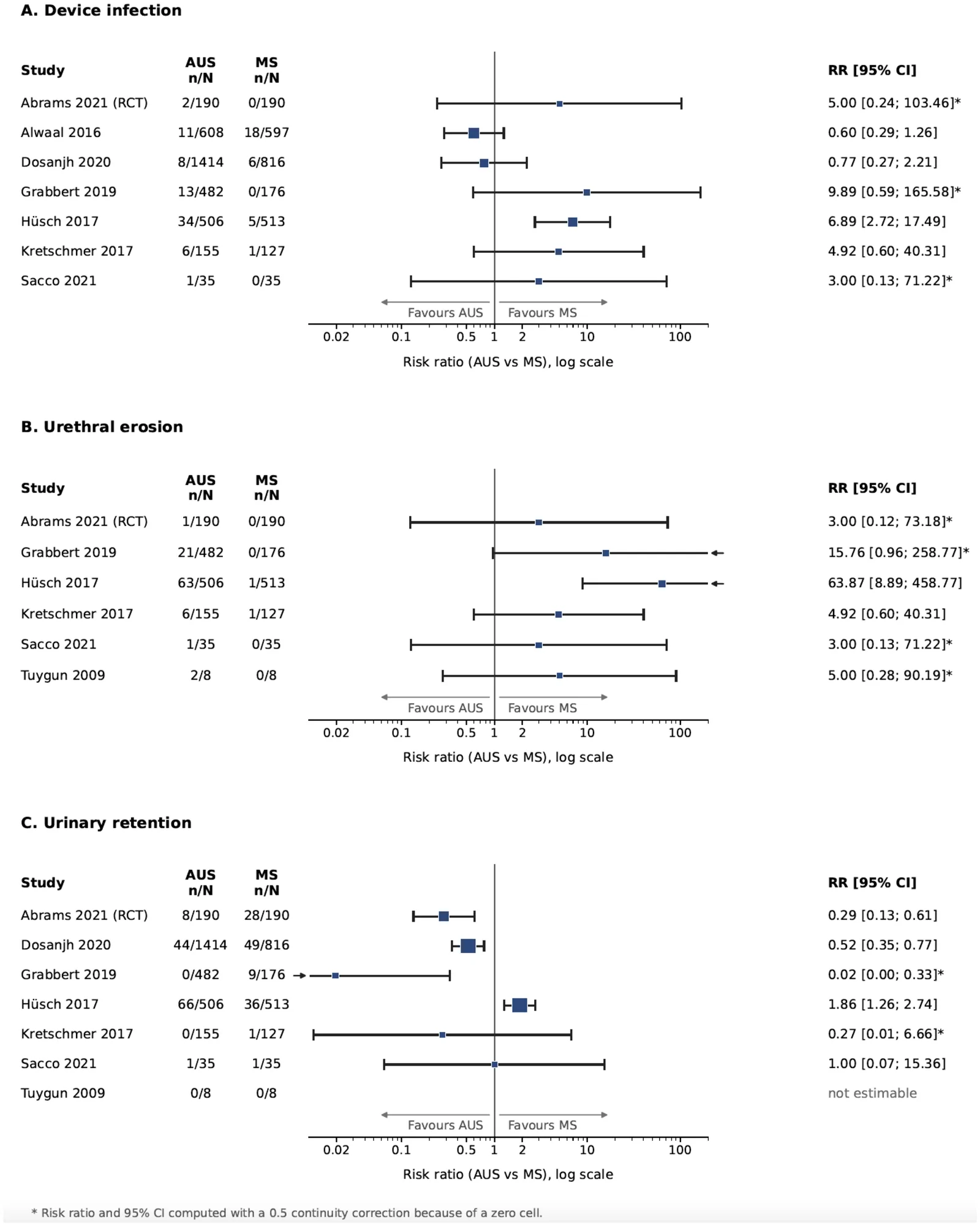
Safety (infection, erosion and retention rates).

- Device infection: 0–3% MS vs. 1–7% AUS
- Urethral erosion: 0–1% MS vs. 1–25% AUS
- Urinary retention: 1–15% MS vs. 0–13% AUS

For device infection, the direction of the within-study comparison was inconsistent: the risk ratio for AUS versus MS was below 1 in the two administrative-database cohorts (0.60, 95% CI 0.29–1.26; and 0.77, 0.27–2.21) and above 1 in the clinical cohorts, reaching significance in one study only (6.89, 2.72–17.49). For urethral erosion, the risk ratio was above 1 in all six studies reporting this outcome (3.00 to 63.87), significantly so in one study (63.87, 8.89–458.77). For urinary retention, the risk ratio was below 1 in four of six estimable studies, including the only randomized trial (0.29, 0.13–0.61) and the largest cohort (0.52, 0.35–0.77), null in one and above 1 in one (1.86, 1.26–2.74). Overall, urethral erosion was more frequent after AUS, urinary retention was more frequent after MS in most studies, and no consistent difference was observed for device infection. Thus, MS generally showed fewer erosions, but more retention than AUS.

Mean follow-up ranged from 1 to 51 months. When studies were grouped by follow-up duration, the direction of the study comparison was unchanged for urethral erosion and urinary retention across all three follow-up categories, whereas the two studies reporting a lower infection risk after AUS were the two with the shortest observation periods.

MS may be safer than AUS for selected patients, but this interpretation should be cautious given the absence of subgroup analyses in irradiated patients and the possibility of severe complications in this population.

## Discussion

This systematic review focuses on comparative studies of AUS versus MS in terms of efficacy and safety. AUS achieved higher efficacy rates, with continence ranging from 14–94% compared with 11–69% for MS. Safety outcomes favored MS, with lower erosion rates, but higher rates of urinary retention. This systematic review is restricted to studies in which both devices were compared within the same cohort. Its findings are consistent in direction with the previous reviews of Chen et al. (13)., Choinière et al. (14), Grigoryan et al. (15) and Lin et al. (16), but differ from them in that no estimate presented here derives from an indirect comparison of separate case series.

These findings align with published data.

In the SATURN registry, continence improved from 49% to 76% at 1 year after AUS and from 17–33% to 37–76% after MS, depending on sling type. Revision rates were higher for AUS (17).

The MASTER trial was the only RCT; at 12 months, incontinence persisted in 87.0% of MS patients and 84.2% of AUS patients. These rates are much higher than in most other studies, underscoring variability and limiting comparability. This study reported a patient-reported measure of persistent incontinence at 12 months rather than a pad-based definition of dryness. The proportions of men meeting the review definition of continence were consequently much lower in that trial than in the observational cohorts, in both arms. This difference is most plausibly attributable to the outcome instrument rather than to a difference in surgical effectiveness, and it illustrates why cross-study comparison of crude proportions is unreliable in this field.

Previous systematic reviews have been limited to specific populations. For post-prostatectomy incontinence, Chen et al. reported cure rates of 60% for both MS and AUS, but heterogeneity prevented direct comparison (13). Choiniere et al. found cure rates of 58.6% for MS and 74.0% for AUS (14). Grigoryan et al. highlighted difficulties in interpretation due to variable definitions and study designs (15). Lin et al, in five studies of moderate incontinence, reported higher success with AUS (OR 0.57, 95% CI 0.36–0.90) (16).

Limitations of our review include the heterogeneity of included populations and outcome definitions.

The principal limitation of this review is confounding by indication. In routine practice, male slings and artificial urinary sphincters are not offered to equivalent populations: slings are preferentially proposed for mild to moderate, non-irradiated incontinence in the primary setting, whereas sphincters are preferentially proposed for severe, irradiated or previously operated incontinence. Baseline severity and prognostic factors were incompletely reported and could not be used for multivariable adjustment, and only one of the nine included studies was randomized and one propensity-score matched. The within-study differences reported here should therefore be read as associations observed in clinically dissimilar groups, not as estimates of the causal effect of one device relative to the other.

We restricted inclusion to comparative studies, ensuring that within each study MS and AUS were evaluated with the same criteria, but precluding cross-study comparisons.

As with other reviews (16, 18, 19), heterogeneity prevented meta-analysis. We also could not perform multivariate adjustment for confounders such as radiation therapy, obesity, age, surgical approach, redo surgery, or baseline incontinence severity. Similar concerns were raised by Van Der Aa et al. (20), who emphasized the need to update AUS outcomes to contemporary evidence-based standards.

Future research should aim to standardize outcome reporting. The FDA has emphasized the importance of the pad weight test (PWT) as a reliable endpoint, but implementation is complex (21). Alternative solutions are needed, such as defining core outcome sets, as proposed in the SATURN initiative.

## Conclusion

Comparative evidence generally suggests higher continence proportions after artificial urinary sphincter implantation than after male sling implantation in the cohorts studied, while the complication profiles of the two devices differ: urethral erosion are more frequent after the sphincter, and postoperative urinary retention is more frequent after the sling. Conclusions regarding device infections are inconsistent. Confidence in these findings is limited by clinical and methodological heterogeneity, by confounding by indication in populations that are not clinically equivalent, by inconsistent outcome definitions and by the scarcity of randomized evidence; the certainty of the evidence was rated low to very low. There is an urgent need for standardized outcome reporting, for a core outcome set, and for better monitoring of device safety and effectiveness; access to national healthcare databases offers new opportunities to strengthen the evidence in this field.

## Data Availability

The original contributions presented in the study are included in the article/**Supplementary Material**. Further inquiries can be directed to the corresponding author.
