# Supplementary material for: ‘Efficacy and safety outcomes after slings or artificial urinary sphincter implantation for non-neurogenic male stress urinary incontinence: a systematic review’ for more clarity
Source: Front Urol. 2026 Oct 2;6:1852334. doi: 10.3389/fruro.2026.1852334 (PMC13662287; doi:10.3389/fruro.2026.1852334)
Supplement: Supplementary file 1 [file DataSheet1.pdf]

Appendix A: Amstar 2

AMSTAR 2: a critical appraisal tool for systematic reviews that include randomised or non-randomised studies of healthcare interventions, or both

1. Did the research questions and inclusion criteria for the review include the components of PICO?

For Yes:	Optional (recommended)	
☐ Population<br>☐ Intervention<br>☐ Comparator group<br>☐ Outcome	☐ Timeframe for follow-up	■ Yes<br>☐ No

2. Did the report of the review contain an explicit statement that the review methods were established prior to the conduct of the review and did the report justify any significant deviations from the protocol?

For Partial Yes:	For Yes:	
The authors state that they had a written protocol or guide that included ALL the following:<br>☐ review question(s)<br>☐ a search strategy<br>☐ inclusion/exclusion criteria<br>☐ a risk of bias assessment	As for partial yes, plus the protocol should be registered and should also have specified:<br>☐ a meta-analysis/synthesis plan, if appropriate, *and*<br>☐ a plan for investigating causes of heterogeneity<br>☐ justification for any deviations from the protocol	■ Yes<br>☐ Partial Yes<br>☐ No

3. Did the review authors explain their selection of the study designs for inclusion in the review?

For Yes, the review should satisfy ONE of the following:	
☐ *Explanation for* including only RCTs<br>☐ OR *Explanation for* including only NRSI<br>☐ OR *Explanation for* including both RCTs and NRSI	■ Yes<br>☐ No

4. Did the review authors use a comprehensive literature search strategy?

For Partial Yes (all the following):	For Yes, should also have (all the following):	
☐ searched at least 2 databases (relevant to research question)<br>☐ provided key word and/or search strategy<br>☐ justified publication restrictions (e.g. language)	☐ searched the reference lists / bibliographies of included studies<br>☐ searched trial/study registries<br>☐ included/consulted content experts in the field<br>☐ where relevant, searched for grey literature<br>☐ conducted search within 24 months of completion of the review	■ Yes<br>☐ Partial Yes<br>☐ No

5. Did the review authors perform study selection in duplicate?

For Yes, either ONE of the following:	
☐ at least two reviewers independently agreed on selection of eligible studies and achieved consensus on which studies to include<br>☐ OR two reviewers selected a sample of eligible studies and achieved good agreement (at least 80 percent), with the remainder selected by one reviewer.	■ Yes<br>☐ No

AMSTAR 2: a critical appraisal tool for systematic reviews that include randomised or non-randomised studies of healthcare interventions, or both

6. Did the review authors perform data extraction in duplicate?

For Yes, either ONE of the following:

- ☐ at least two reviewers achieved consensus on which data to extract from included studies
- ☐ OR two reviewers extracted data from a sample of eligible studies and achieved good agreement (at least 80 percent), with the remainder extracted by one reviewer.

■ Yes
☐ No

7. Did the review authors provide a list of excluded studies and justify the exclusions?

For Partial Yes:	For Yes, must also have:	
☐ provided a list of all potentially relevant studies that were read in full-text form but excluded from the review	☐ Justified the exclusion from the review of each potentially relevant study	■ Yes<br>☐ Partial Yes<br>☐ No

8. Did the review authors describe the included studies in adequate detail?

For Partial Yes (ALL the following):	For Yes, should also have ALL the following:	
☐ described populations<br>☐ described interventions<br>☐ described comparators<br>☐ described outcomes<br>☐ described research designs	☐ described population in detail<br>☐ described intervention in detail (including doses where relevant)<br>☐ described comparator in detail (including doses where relevant)<br>☐ described study's setting<br>☐ timeframe for follow-up	■ Yes<br>☐ Partial Yes<br>☐ No

9. Did the review authors use a satisfactory technique for assessing the risk of bias (RoB) in individual studies that were included in the review?

**RCTs**<br>For Partial Yes, must have assessed RoB from<br>☐ unconcealed allocation, *and*<br>☐ lack of blinding of patients and assessors when assessing outcomes (unnecessary for objective outcomes such as all-cause mortality)	For Yes, must also have assessed RoB from:<br>☐ allocation sequence that was not truly random, *and*<br>☐ selection of the reported result from among multiple measurements or analyses of a specified outcome	■ Yes<br>☐ Partial Yes<br>☐ No<br>☐ Includes only NRSI
**NRSI**<br>For Partial Yes, must have assessed RoB:<br>☐ from confounding, *and*<br>☐ from selection bias	For Yes, must also have assessed RoB:<br>☐ methods used to ascertain exposures and outcomes, *and*<br>☐ selection of the reported result from among multiple measurements or analyses of a specified outcome	■ Yes<br>☐ Partial Yes<br>☐ No<br>☐ Includes only RCTs

10. Did the review authors report on the sources of funding for the studies included in the review?

For Yes

- ☐ Must have reported on the sources of funding for individual studies included in the review. Note: Reporting that the reviewers looked for this information but it was not reported by study authors also qualifies

■ Yes
☐ No

11. If meta-analysis was performed did the review authors use appropriate methods for statistical combination of results?

For Yes:	
**RCTs**<br>For Yes:<br>☐ The authors justified combining the data in a meta-analysis<br>☐ AND they used an appropriate weighted technique to combine study results and adjusted for heterogeneity if present.<br>☐ AND investigated the causes of any heterogeneity	■ Yes<br>☐ No<br>☐ No meta-analysis conducted
**For NRSI**<br>For Yes:<br>☐ The authors justified combining the data in a meta-analysis<br>☐ AND they used an appropriate weighted technique to combine study results, adjusting for heterogeneity if present<br>☐ AND they statistically combined effect estimates from NRSI that were adjusted for confounding, rather than combining raw data, or justified combining raw data when adjusted effect estimates were not available<br>☐ AND they reported separate summary estimates for RCTs and NRSI separately when both were included in the review	■ Yes<br>☐ No<br>☐ No meta-analysis conducted

12. If meta-analysis was performed, did the review authors assess the potential impact of RoB in individual studies on the results of the meta-analysis or other evidence synthesis?

For Yes:<br>☐ included only low risk of bias RCTs<br>☐ OR, if the pooled estimate was based on RCTs and/or NRSI at variable RoB, the authors performed analyses to investigate possible impact of RoB on summary estimates of effect.	■ Yes<br>☐ No<br>☐ No meta-analysis conducted

13. Did the review authors account for RoB in individual studies when interpreting/ discussing the results of the review?

For Yes:<br>☐ included only low risk of bias RCTs<br>☐ OR, if RCTs with moderate or high RoB, or NRSI were included the review provided a discussion of the likely impact of RoB on the results	■ Yes<br>☐ No

14. Did the review authors provide a satisfactory explanation for, and discussion of, any heterogeneity observed in the results of the review?

For Yes:<br>☐ There was no significant heterogeneity in the results<br>☐ OR if heterogeneity was present the authors performed an investigation of sources of any heterogeneity in the results and discussed the impact of this on the results of the review	■ Yes<br>☐ No

15. If they performed quantitative synthesis did the review authors carry out an adequate investigation of publication bias (small study bias) and discuss its likely impact on the results of the review?

For Yes:<br>☐ performed graphical or statistical tests for publication bias and discussed the likelihood and magnitude of impact of publication bias	■ Yes<br>☐ No<br>☐ No meta-analysis conducted

AMSTAR 2: a critical appraisal tool for systematic reviews that include randomised or non-randomised studies of healthcare interventions, or both

16. Did the review authors report any potential sources of conflict of interest, including any funding they received for conducting the review?

For Yes:	
☐ The authors reported no competing interests OR	■ Yes
☐ The authors described their funding sources and how they managed potential conflicts of interest	☐ No

To cite this tool: Shea BJ, Reeves BC, Wells G, Thuku M, Hamel C, Moran J, Moher D, Tugwell P, Welch V, Kristjansson E, Henry DA. AMSTAR 2: a critical appraisal tool for systematic reviews that include randomised or non-randomised studies of healthcare interventions, or both. BMJ. 2017 Sep 21;358:j4008.

Section and Topic	Item #	Checklist item	Location where item is reported
**TITLE**			
Title	1	Identify the report as a systematic review.	Title
**ABSTRACT**			
Abstract	2	See the PRISMA 2020 for Abstracts checklist.	1
**INTRODUCTION**			
Rationale	3	Describe the rationale for the review in the context of existing knowledge.	2
Objectives	4	Provide an explicit statement of the objective(s) or question(s) the review addresses.	3
**METHODS**			
Eligibility criteria	5	Specify the inclusion and exclusion criteria for the review and how studies were grouped for the syntheses.	4
Information sources	6	Specify all databases, registers, websites, organisations, reference lists and other sources searched or consulted to identify studies. Specify the date when each source was last searched or consulted.	3
Search strategy	7	Present the full search strategies for all databases, registers and websites, including any filters and limits used.	3
Selection process	8	Specify the methods used to decide whether a study met the inclusion criteria of the review, including how many reviewers screened each record and each report retrieved, whether they worked independently, and if applicable, details of automation tools used in the process.	5
Data collection process	9	Specify the methods used to collect data from reports, including how many reviewers collected data from each report, whether they worked independently, any processes for obtaining or confirming data from study investigators, and if applicable, details of automation tools used in the process.	5
Data items	10a	List and define all outcomes for which data were sought. Specify whether all results that were compatible with each outcome domain in each study were sought (e.g. for all measures, time points, analyses), and if not, the methods used to decide which results to collect.	5
	10b	List and define all other variables for which data were sought (e.g. participant and intervention characteristics, funding sources). Describe any assumptions made about any missing or unclear information.	5
Study risk of bias assessment	11	Specify the methods used to assess risk of bias in the included studies, including details of the tool(s) used, how many reviewers assessed each study and whether they worked independently, and if applicable, details of automation tools used in the process.	6
Effect measures	12	Specify for each outcome the effect measure(s) (e.g. risk ratio, mean difference) used in the synthesis or presentation of results.	6
Synthesis methods	13a	Describe the processes used to decide which studies were eligible for each synthesis (e.g. tabulating the study intervention characteristics and comparing against the planned groups for each synthesis (item #5)).	6
	13b	Describe any methods required to prepare the data for presentation or synthesis, such as handling of missing summary statistics, or data conversions.	6
	13c	Describe any methods used to tabulate or visually display results of individual studies and syntheses.	6
	13d	Describe any methods used to synthesize results and provide a rationale for the choice(s). If meta-analysis was performed, describe the model(s), method(s) to identify the presence and extent of statistical heterogeneity, and software package(s) used.	6

Section and Topic	Item #	Checklist item	Location where item is reported
	13e	Describe any methods used to explore possible causes of heterogeneity among study results (e.g. subgroup analysis, meta-regression).	6
	13f	Describe any sensitivity analyses conducted to assess robustness of the synthesized results.	6
Reporting bias assessment	14	Describe any methods used to assess risk of bias due to missing results in a synthesis (arising from reporting biases).	6
Certainty assessment	15	Describe any methods used to assess certainty (or confidence) in the body of evidence for an outcome.	6
**RESULTS**			
Study selection	16a	Describe the results of the search and selection process, from the number of records identified in the search to the number of studies included in the review, ideally using a flow diagram.	6
	16b	Cite studies that might appear to meet the inclusion criteria, but which were excluded, and explain why they were excluded.	6
Study characteristics	17	Cite each included study and present its characteristics.	7
Risk of bias in studies	18	Present assessments of risk of bias for each included study.	7
Results of individual studies	19	For all outcomes, present, for each study: (a) summary statistics for each group (where appropriate) and (b) an effect estimate and its precision (e.g. confidence/credible interval), ideally using structured tables or plots.	8
Results of syntheses	20a	For each synthesis, briefly summarise the characteristics and risk of bias among contributing studies.	6
	20b	Present results of all statistical syntheses conducted. If meta-analysis was done, present for each the summary estimate and its precision (e.g. confidence/credible interval) and measures of statistical heterogeneity. If comparing groups, describe the direction of the effect.	7
	20c	Present results of all investigations of possible causes of heterogeneity among study results.	8
	20d	Present results of all sensitivity analyses conducted to assess the robustness of the synthesized results.	8
Reporting biases	21	Present assessments of risk of bias due to missing results (arising from reporting biases) for each synthesis assessed.	8
Certainty of evidence	22	Present assessments of certainty (or confidence) in the body of evidence for each outcome assessed.	8
**DISCUSSION**			
Discussion	23a	Provide a general interpretation of the results in the context of other evidence.	9
	23b	Discuss any limitations of the evidence included in the review.	9
	23c	Discuss any limitations of the review processes used.	10
	23d	Discuss implications of the results for practice, policy, and future research.	10
**OTHER INFORMATION**			
Registration and	24a	Provide registration information for the review, including register name and registration number, or state that the review was not registered.	3

Section and Topic	Item #	Checklist item	Location where item is reported
protocol	24b	Indicate where the review protocol can be accessed, or state that a protocol was not prepared.	3
	24c	Describe and explain any amendments to information provided at registration or in the protocol.	3
Support	25	Describe sources of financial or non-financial support for the review, and the role of the funders or sponsors in the review.	Title
Competing interests	26	Declare any competing interests of review authors.	Title
Availability of data, code and other materials	27	Report which of the following are publicly available and where they can be found: template data collection forms; data extracted from included studies; data used for all analyses; analytic code; any other materials used in the review.	Title

From: Page MJ, McKenzie JE, Bossuyt PM, Boutron I, Hoffmann TC, Mulrow CD, et al. The PRISMA 2020 statement: an updated guideline for reporting systematic reviews. BMJ 2021;372:n71. doi: 10.1136/bmj.n71. This work is licensed under CC BY 4.0. To view a copy of this license, visit https://creativecommons.org/licenses/by/4.0/

Appendix B: PRISMA statement

Appendix C: Search Algorithm

Medline & Cochrane

1. Urinary Incontinence [mh]
2. “Urinary incontinence” [tiab]
3. Suburethral Slings [mh]
4. “Suburethral slings” [tiab]
5. “Suburethral tapes” [tiab]
6. “Suburethral mesh” [tiab]
7. “Midurethral slings” [tiab]
8. “Midurethral tapes” [tiab]
9. “Midurethral mesh” [tiab]
10. “Suburethral sling” [tiab]
11. “Suburethral tape” [tiab]
12. “Midurethral sling” [tiab]
13. “Midurethral tape” [tiab]
14. Urinary Sphincter, Artificial [mh]
15. “Artificial Urinary Sphincter” [tiab]
16. “Artificial Urinary Sphincters” [tiab]
17. Male [mh]
18. “Men” [tiab]
19. 1 OR 2
20. 3 OR 4 5 OR 6 OR 7 OR 8 OR 9 OR 10 OR 11 OR 12 OR 13
21. 14 OR 15 OR 16
22. 17 OR 18
23. 19 AND 20 AND 21 AND 22

Appendix D:

CASP Cohort Study Checklist
(URL: https://casp-uk.b-cdn.net/wpa-content/uploads/2018/01/CASP-Cohort-Study-Checklist_2018.pdf)

Section A: Are the results of the study valid?
1. Did the study address a clearly focused issue?
2. Was the cohort recruited in an acceptable way?
3. Was the exposure accurately measured to minimize bias?
4. Was the outcome accurately measured to minimize bias?
5. (a) Have the authors identified all important confounding factors?
5. (b) Have they take account of the confounding factors in the design and/or analysis?
6. (a) Was the follow up of subjects complete enough?
6. (b) Was the follow up of subjects long enough?

Section B: What are the results?
7. What are the results of this study?
8. How precise are the results?
9. Do you believe the results?

Section C: Will the results help locally?
10. Can the results be applied to the local population?
11. Do the results of this study fit with other available evidence?
12. What are the implications of this study for practice?

Year	Studies	1	2	3	4	5a	5b	6a	6b	7	8	9	10	11	12
2015	Ajay	Y	Y	Y	Y	Y	Y	Y	N	Treatment failure was seen in 55% with a sling vs 6% with an artificial urinary sphincter.	high	Y	Y	Y	Y
2016	Alwaal	Y	Y	Y	Y	Y	Y	Y	N	Male sling is associated with a lower rate of complication rate compared to AUS (2.8 vs 5.1 %, p = 0.046).	high	Y	Y	Y	Y
2019	Dosanjh	Y	Y	Y	Y	Y	Y	Y	Y	There is a volume effect for outcomes of AUS procedures, suggesting that they should only be performed in high volume centres.	mid	Y	Y	Y	Y
2018	Grabbert	Y	Y	Y	Y	Y	Y	Y	N	At maximum follow-up, patients that underwent an AUS implantation had a significantly lower mean pad usage during daytime (p < 0.001) and nighttime (p = 0.018).	high	Y	Y	Y	Y
2016	Hush	Y	Y	Y	Y	Y	Y	Y	Y	A history of pelvic irradiation was an independent risk factor for explantation in AUS (p < 0.001) and MS (p = 0.018).	high	Y	Y	Y	Y
2017	Kretshmer	Y	Y	Y	Y	Y	Y	Y	N	Increased intraoperative complication rates after adjustable male sling implantation (15.9% [adjustable male sling] vs. 4.2% [AUS], P=0.003).	high	Y	Y	Y	Y
2020	Sacco	Y	Y	Y	Y	Y	Y	Y	Y	At the last follow-up, the median 24-h leakage volumes, median ICIQ-SF scores and satisfaction rates were 0 vs 15 mL (P = 0.017), 4 vs 10 (P = 0.001), and 94.3% vs 68.6% (P = 0.012) in the AUS and RTS cohorts, respectively.	high	Y	Y	Y	Y
2009	Tuygun	Y	Y	Y	Y	Y	Y	Y	N	The results have shown that AUS implantation results in better outcomes than placement of the adjustable bulbourethral male sling as secondary therapy	mid	Y	Y	Y	Y

Appendix E: Risk of Bias Tool

Unique ID	Experimental	Comparator	Outcome	D1	D2	D3	D4	D5	Overall
Abrams	MS	AUS	efficacy	+	+	+	!	+	!

+	Low risk
!	Some concerns
-	High risk

D1	Randomisation process
D2	Deviations from the intended interventions
D3	Missing outcome data
D4	Measurement of the outcome
D5	Selection of the reported result
